# Intracerebroventricular administration of lipopolysaccharide induces indoleamine-2,3-dioxygenase-dependent depression-like behaviors

**DOI:** 10.1186/1742-2094-10-87

**Published:** 2013-07-18

**Authors:** Marcus A Lawson, Jennifer M Parrott, Robert H McCusker, Robert Dantzer, Keith W Kelley, Jason C O’Connor

**Affiliations:** 1 Neuroscience Program, University of Illinois at Urbana-Champaign, Urbana, IL 61801, USA; 2 Integrative Immunology and Behavior Program, Department of Animal Sciences, University of Illinois at Urbana-Champaign, Urbana, IL 61801, USA; 3 Department of Pharmacology, University of Texas Health Science Center at San Antonio, 216B Medical Building MC-7764, 7703 Floyd Curl Drive, San Antonio, TX 78229-3900, USA; 4 Center for Biomedical Neuroscience, University of Texas Health Science Center at San Antonio, 216B Medical Building MC-7764, 7703 Floyd Curl Drive, San Antonio, TX 78229-3900, USA; 5 Department of Pathology, University of Illinois at Urbana-Champaign, Urbana, IL 61801, USA; 6 MD Anderson Cancer Center, Division of Internal Medicine, Department of Symptom Research, Houston, TX 77030, USA; 7 Mood Disorders Translational Research Core, University of Texas Health Science Center at San Antonio, 216B Medical Building MC-7764, 7703 Floyd Curl Drive, San Antonio, TX 78229-3900, USA

## Abstract

**Background:**

Activation of the tryptophan degrading enzyme indoleamine-2,3-dioxygenase 1 (IDO1) is associated with the development of behavioral signs of depression. Systemic immune challenge induces IDO1 in both the periphery and the brain, leading to increased circulating and brain concentrations of kynurenines. However, whether IDO1 activity within the brain is necessary for the manifestation of depression-like behavior of mice following a central immune challenge remains to be elucidated.

**Methods:**

We investigated the role of brain IDO1 in mediating depression-like behavior of mice in response to intracerebroventricular injection of saline or lipopolysaccharide (LPS, 10 ng).

**Results:**

LPS increased the duration of immobility in the tail suspension test and decreased preference for a sucrose solution. These effects were associated with an activation of central but not peripheral IDO1, as LPS increased brain kynurenine but had no effect on plasma concentrations of kynurenine. Interestingly, genetic deletion or pharmacological inhibition of IDO1, using 1-methyl-tryptophan, abrogated the reduction in sucrose preference induced by intracerebroventricular LPS. 1-Methyl-tryptophan also blocked the LPS-induced increase in duration of immobility during the tail suspension test.

**Conclusions:**

These data indicate that activation of brain IDO1 is sufficient to induce depression-like behaviors of mice in response to central LPS.

## Background

Over the past several decades, a link between inflammation and neuropsychiatric disorders has been firmly established at both the clinical and preclinical levels [1]. In depressed patients, many studies have reported an association between elevated levels of peripheral inflammatory markers (for example, plasma IL-6 and C-reactive protein) and symptoms of major depression [2,3]. Also, peripheral inflammation induced by lipopolysaccharide (LPS), in both human volunteers and rodent models, precipitates significant changes in cognitive function, mood and behavior [4-7]. Individuals suffering from neurodegenerative diseases with a neuroinflammatory signature, such as multiple sclerosis [8], Huntington’s disease [9], Parkinson’s disease [10] and Alzheimer’s disease [11], also have increased prevalence of comorbid neuropsychiatric disturbances. A common factor in these processes is the increased expression of proinflammatory cytokines in the brain that may mediate the core neuropsychiatric and neurovegetative symptoms of major depression [12].

Cytokines released within the brain can directly impact neuron function, as evidenced by IL-1β-induced and TNFα-induced changes in long-term potentiation [13-15], but they can also act indirectly by stimulating the production of neuroactive molecules that have been associated with depression. In the last decade, increasing interest has focused on the tryptophan degrading enzyme indoleamine-2,3-dioxygenase 1 (IDO1). During inflammation, IDO1 is the first and rate-limiting enzyme in a metabolic cascade leading to increased levels of kynurenine in the circulation and tissues. Moreover, increased IDO1 enzymatic activity and elevated kynurenine concentration levels have been correlated with inflammation-associated depression [16-19]. Recent preclinical research has demonstrated that pharmacological inhibition of IDO1 enzymatic activity or genetic deletion of IDO1 abrogates inflammation-dependent behavioral changes that model depression. This has been demonstrated in a murine model of acute inflammation induced by peripheral LPS and in a model of chronic inflammation induced by peripheral infection with Bacille Calmette Guérin [7,20-22]. There is already evidence that direct activation of central cytokine signaling pathways by intracerebroventricular (ICV) administration of TNFα, LPS or the human immunodeficiency virus transactivator of transcription (Tat) can precipitate the development of depression-like behavior in rodents that is associated with upregulation of cytokines and IDO1 [23-25]. However, the causal role of brain IDO1 activation in these models has not yet been established.

To explore the potential of brain kynurenine metabolism in driving inflammation-induced depression-like behavior, we challenged mice with a single ICV injection of LPS. Central LPS precipitated depression-like behaviors coupled with increased kynurenine concentrations and kynurenine:tryptophan ratio specifically within the brain. In contrast, IDO1 knockout (KO) or wild-type (WT) mice pretreated with 1-methyl-tryptophan (1MT) were protected from developing LPS-induced depression-like behavior compared with LPS-treated WT controls. Taken together, these data indicate that upregulation of brain IDO1 is sufficient for the development of depression-like behaviors following ICV LPS.

## Methods

### Animals

Male Balb/C mice (Charles Rivers Laboratories, Wilmington, MA, USA), C57BL/6J (WT) mice and IDO1 homozygous deficient (KO) mice (The Jackson Laboratory, Bar Harbor, ME, USA) were individually housed and provided with *ad libitum* access to chow (Teklad 8640, Harlan laboratories, Indianapolis, IN, USA) and water. Mice were housed in a temperature-controlled and humidity-controlled room maintained on a 12-hour reverse light/dark cycle. Mice were allowed to acclimate to these conditions for at least 7 days before being implanted with a guide cannula for mice (Plastics One, Roanoke, VA, USA) placed stereotaxically to extend 1 mm dorsal to the lateral ventricle, as previously described [25]. Cannuli were placed at 1.5 mm lateral, 0.6 mm posterior, and 1.3 mm dorsal with respect to bregma. Guide cannulas were kept clean and covered using a screw-on dummy cannula (Plastics One). Mice were given 10 to 14 days to recover from surgery prior to treatment. All procedures performed on mice were in compliance with the National Institutes of Health guidelines and were approved by the Institutional Animal Care and Use Committees at both the University of Texas Health Science Center at San Antonio and the University of Illinois at Urbana-Champaign.

Treatments were administered at the onset of the dark cycle. ICV injections were administered using a 10 μl gas-tight syringe attached to internal injector cannulas (Plastics One) that extended 1 mm beyond the tip of the guide cannula, thus penetrating the lateral ventricle. All mice received treatments in 1 μl injection volume over a 1-minute time period followed by an additional 1-minute delay to allow diffusion before removing the injector cannula. Mice were injected ICV with either saline (saline) or with LPS (10 ng/μl) from *Escherichia coli* O127:B8 (Sigma Aldrich, St Louis, MO, USA) prepared in saline. This dose of LPS has been previously demonstrated to induce a transient sickness behavior response in multiple strains of mice that is resolved by 24 hours post administration, and the dose was selected based on its ability to induce depression-like behaviors that are temporally distinct from the acute sickness behaviors [26-29].

### Experiment 1: Intracerebroventricular administration of LPS precipitates depressive-like behavior and upregulates kynurenine metabolism specifically within the brain

Experiment 1 was performed in Balb/C mice to determine whether direct activation of the central innate immune response was sufficient to trigger the development of depression-like behaviors and to establish whether ICV LPS increased kynurenine pathway metabolism specifically within the brain, as measured by the kynurenine:tryptophan ratio. Twenty-four hours after receiving either saline or LPS administration into the lateral ventricles, depression-like behaviors were measured. After behavioral testing, mice were rapidly euthanized in a carbon dioxide chamber and laparotomized to facilitate blood collection and vascular perfusion. Blood samples were collected from the inferior vena cava. Immediately following blood collection, the chest was opened, a knick was made in the right atrium and ~30 ml ice-cold heparinized saline was perfused via the left ventricle. Brains were then rapidly removed and placed in a vial on dry ice. The brains were stored at −80°C until processing. Brains were removed from −80°C storage and pulverized with a ceramic mortar and pestle chilled on dry ice to maintain the brains in a frozen state. The brain powder was mixed to homogenize the tissue and the powder was then aliquoted for analysis. The purpose of this procedure was to divide the tissue equally and negate potential effects of hemispheric differences brought about by cannulation.

#### Depression-like behavior

Two tests of depression-like behavior were utilized in each study [30]. At 24 hours after treatment, mice were submitted to the tail suspension test (TST) using the Mouse Tail Suspension Package (Med Associates, St Albans, VT, USA) as previously described [31]. During the TST, mice were suspended by their tail using adhesive medical tape attached to a strain force gauge. Mice were tested for 10 minutes and were considered immobile when the force was below a lower threshold that was determined for each individual mouse.

To determine whether mice display signs of anhedonic behavior, we utilized a two-bottle sucrose preference test. Mice were given *ad libitum* access to drink from bottles containing either water or 1% (C57BL6/J) or 2% (Balb/C) sucrose solution for a 24-hour period. Immediately prior to being placed on the home cage (immediately after treatment) and 24 hours later, the bottles were weighed so that the amount of each solution that was consumed could be calculated. Preference was calculated by determining the percentage of sucrose consumed divided by the total fluid intake (sucrose intake/total fluid intake × 100).

#### HPLC methods

Homogenized brain and plasma samples were analyzed for kynurenine and tryptophan using a Coulochem III electrochemical detector paired with a model 5041 amperometric analytical cell fitted with a glassy carbon target electrode (Thermo Scientific Dionex, Bannockburn, IL, USA), as previously described [7]. The mobile phase (pH 4.6) consisted of 75 mM NaH_2_PO_4_, 25 μM ethylenediamine tetraacetic acid, and 100 μl/l triethylamine mixed into acetonitrile:water (6:94 v:v). Compounds of interest were separated with a Hypersil ODS C18 analytical column (2.1 mm × 150 mm, 3 μM; Thermo Scientific, West Palm Beach, FL, USA) and compared against an external standard curve made fresh on each day of analysis. Chromatograms were collected and analyzed using EZ Chrom SI software (Agilent Technologies, Santa Clara, CA, USA).

### Experiment 2: Utilization of conventional genetic knockout mice to examine the mechanistic role of IDO1 in ICV LPS-induced depression-like behaviors

Results from Experiment 1 demonstrated a clear association between depression-like behaviors and brain kynurenine metabolism following ICV LPS. To explore the potential mechanistic role of IDO1 in mediating the depression-like behavioral response to LPS, IDO1 KO mice or their WT background strain controls (C57BL/6J) were treated as described in Experiment 1. Depression-like behaviors were also measured following the same protocols as described above. To confirm temporal dissociation between the acute sickness behavior response and depression-like behaviors 24 hours after ICV LPS, an open field test was utilized to assess exploratory locomotor activity, followed by perfusion and tissue collection to measure steady-state mRNA expression of proinflammatory cytokines within the brain.

#### Exploratory locomotor activity

Exploratory locomotor activity, measured as the distance traveled by each mouse within a testing chamber, is a preferred assessment of acute sickness behavior because it reflects the mouse’s capacity and willingness to move freely and to explore a novel environment. Additionally, exploratory locomotor activity correlates tightly to LPS-induced fever responses [32] and does not involve a compensatory phase, such as recovery of lost body weight. The open field chamber consisted of a 40 cm (width) × 40 cm (length) × 30 cm (height) acrylic box with opaque walls. The chamber floor was dimly illuminated from overhead (3 to 10 lux). A 5-minute testing session was video recorded, and the chamber was cleaned with 70% isopropanol between mice. Video files were analyzed using Ethovision XT 7 tracking software (Noldus, Leesburg, VA, USA).

#### RNA isolation and real-time RT-PCR

RNA was isolated using the PureLink® RNA Mini Kit (Life Technologies, Grand Island, NY, USA) according to the manufacturer instructions. RNA purity and concentration was measured spectrophotometrically using a NanoVue spectrophotometer (GE Healthcare Biosciences, Piscataway, NJ, USA). Reverse transcription was carried out on 2 μg total RNA using a high-capacity cDNA RT kit (Life Technologies) according to the manufacturer’s instructions. Real-time RT-PCR was performed over 40 cycles using a CFX384™ Real-Time PCR Detection System (Bio-Rad, Hercules, CA, USA) and prevalidated Taqman® Gene Expression Assays (Life Technologies) for both target and housekeeping control genes: GAPDH (Mm99999915_g1), IL-1β (Mm01336189_m1), TNFα (Mm00443258_m1), and IL-6 (Mm00446190_m1). Data are expressed as the relative fold change using the 2^–ΔΔCt^ calculation method as previously described [33].

### Experiment 3: Effect of 1-methyl tryptophan on ICV LPS-induced depression-like behaviors

To confirm the genotype × treatment interactions that were apparent in Experiment 2, WT mice were administered either (A) 1 μl saline, (B) 1 μg/μl 1MT, (C) 10 ng/μl LPS or (D) a solution containing 1 μg/μl 1MT and 10 ng/ml LPS. 1MT was prepared by dissolving in 1 N HCl, then buffered using sodium hydroxide to pH 6.5 before being diluted to the final treatment concentration in physiological saline. The dose of 1MT used was based on the average brain 1MT concentrations in mice implanted with a subcutaneous chronic release pellet [7,34]. Depression-like behaviors were measured 24 hours post LPS as described in Experiments 1 and 2.

### Statistical analysis

Data were analyzed using Statview 5.0 statistical software package (SAS Institute Inc., Cary, NC, USA) and are represented as the mean ± standard error of the mean. All measures were analyzed using a one-way or a two-way analysis of variance in IDO KO and 1MT studies. When the two-way interaction was significant (*P* <0.05), *post-hoc* analysis using Fisher’s protected least-significant difference test was employed to test for differences among means.

## Results

### Intracerebroventricular administration of LPS precipitates the development of depression-like behaviors in mice (Experiment 1A)

**Figure 1:**
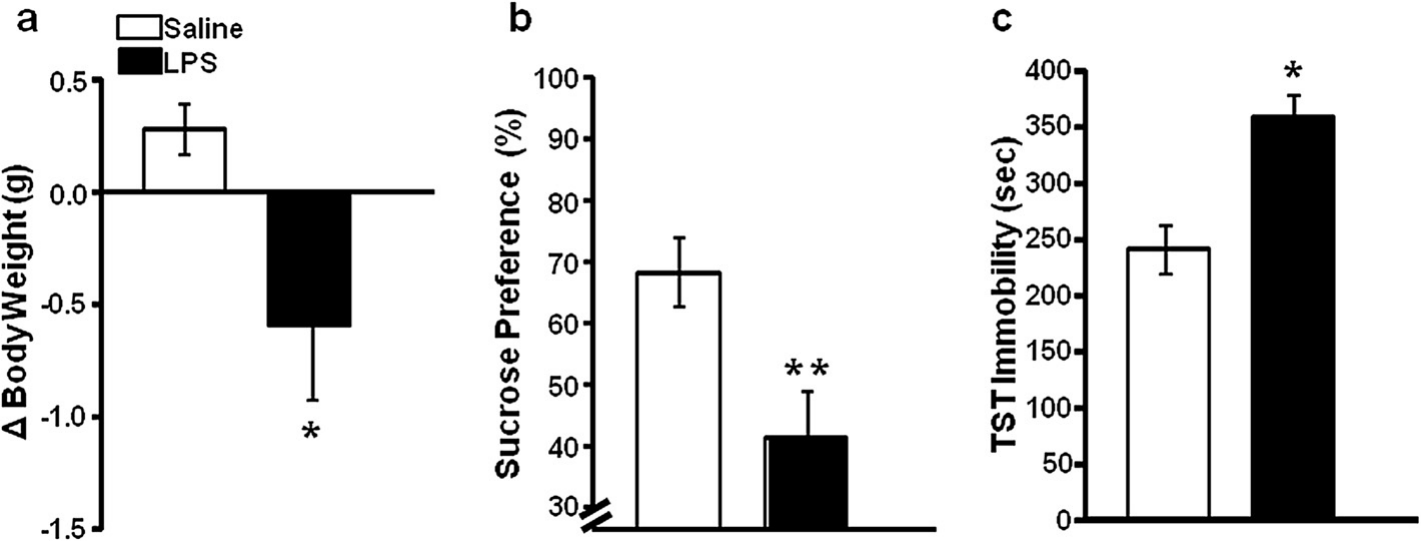
**Lipopolysaccharide administered via intracerebroventricular injection induced body weight loss and depression-like behavior.** (**a**) Lipopolysaccharide (LPS; 10 ng) decreased body weight over a 24-hour period following treatment. (**b**) Sucrose preference was decreased by intracerebroventricular LPS. (**c**) LPS increased tail suspension test (TST) immobility. Data are the average ± standard error of the mean. *P <0.05, **P <0.01. *n* = 7 mice per group.

To confirm that central LPS induced a sickness response, we measured the change in body weight. As expected, Balb/c mice injected ICV with 10 ng LPS lost body weight over a 24-hour period following treatment (*F*_1,12_ = 5.63, *P* <0.05; Figure 1a). We further evaluated whether central LPS induced depression-like behaviors. Sucrose preference was measured during the 24-hour period following treatment. LPS-treated mice displayed a significant decrease in sucrose preference (*F*_1,12_ = 8.31, *P* <0.05; Figure 1b). Mice were then submitted to the TST 24 hours after treatment. LPS caused a significant increase in the duration of immobility when compared with saline-treated control mice (*F*_1,12_ = 16.18, *P* <0.01; Figure 1c).

### Kynurenine to tryptophan ratio is increased in brain, but not plasma, following ICV LPS treatment (Experiment 1B)

To determine whether LPS-induced depression-like behavior was associated with increased levels of kynurenine or an elevated kynurenine:tryptophan ratio, plasma and whole brain samples were collected and analyzed. LPS-treated mice exhibited elevated brain kynurenine concentrations compared with saline-treated mice (*F*_1,11_ = 10.24, *P* <0.01; Table 1), but there was no effect of LPS on brain tryptophan concentrations. Further, elevated brain kynurenine drove an increase in the kynurenine:tryptophan ratio (*F*_1,11_ = 11.26, *P* <0.01). In contrast, LPS treatment had no effect on the plasma kynurenine concentration, tryptophan concentration or kynurenine:tryptophan ratio compared with plasma from saline-treated mice (Table 1).

**Table 1:** Kynurenine and tryptophan 24 hours after treatment

	**Control**	**Lipopolysaccharide**
Brain		
Kynurenine (nmol/mg wet weight)	0.09 ± 0.03^A^	**0.26 ± 0.04**^**B**^
Tryptophan (nmol/mg wet weight)	1.16 ± 0.27^A^	1.45 ± 0.09^A^
Kynurenine:tryptophan	0.07 ± 0.02^A^	**0.19 ± 0.03**^**B**^
Plasma		
Kynurenine (μmol/l)	0.89 ± 0.1^A^	0.99 ± 0.1^A^
Tryptophan (μmol/l)	44.27 ± 1.84^A^	47.63 ± 3.46^A^
Kynurenine:tryptophan	0.020 ± 0.002^A^	0.021 ± 0.003^A^

Brain and plasma were collected for analysis of kynurenine and tryptophan concentrations using HPLC. Data are average concentrations ± standard error of the mean. Averages within rows with different uppercase superscript letters are significantly different; *P* <0.05. *n* = 6 to 7 mice per group.

### IDO1 knockout mice maintain sucrose preference following ICV LPS (Experiment 2A)

**Figure 2:**
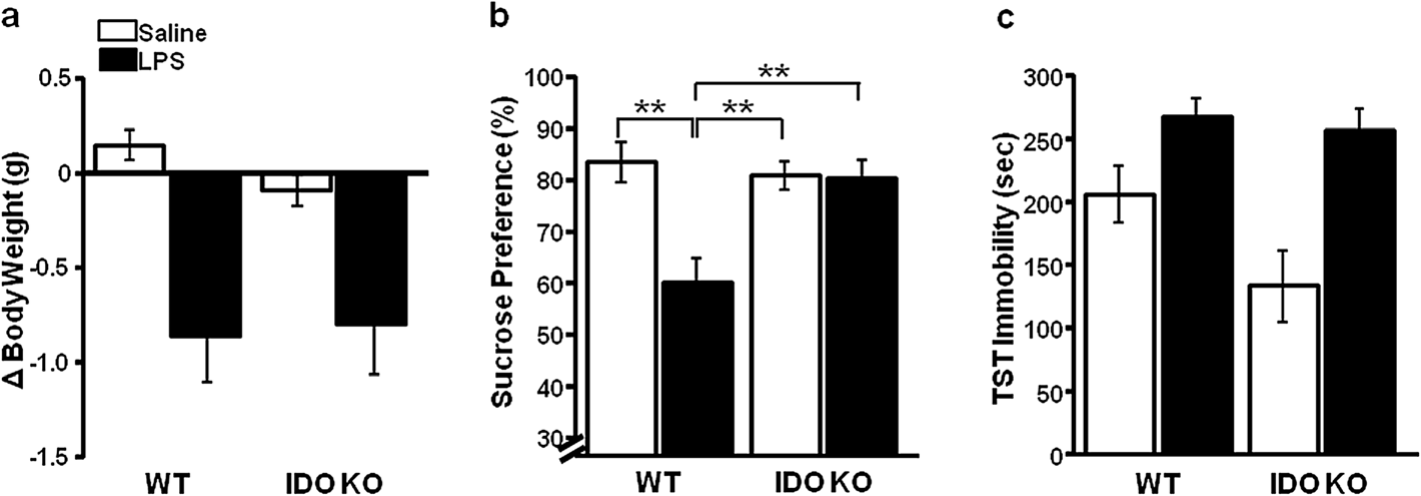
**Mice deficient in indoleamine-2,3-dioxygenase are protected from intracerebroventricular lipopolysaccharide-induced anhedonia.** (**a**) Both wild-type (WT) and indoleamine-2,3-dioxygenase (IDO1) knockout (KO) mice had similar decreases in body weight in response to intracerebroventricular (ICV) lipopolysaccharide (LPS, 10 ng). (**b**) IDO1 KO mice maintained sucrose preference following ICV LPS treatment, but (**c**) had similarly increased tail suspension test (TST) immobility as WT mice. Data are average ± standard error of the mean. **P <0.01. *n* = 6 to 12 mice per group.

To determine whether IDO1 activity is required for central LPS to induce depression-like behavior, we examined whether IDO1 KO mice or WT control mice develop depression-like behavior. Similar to WT mice, IDO1 KO mice experienced a reduction in body weight following ICV LPS treatment (LPS main effect; *F*_1,45_ = 19.7, *P* <0.01; Figure 2a). However, while LPS precipitated a significant 25% reduction in sucrose preference in WT mice, IDO1 KO mice were entirely resistant to the anhedonic effects of ICV LPS, compared with controls (strain × LPS interaction; *F*_3,44_ = 9.18, *P* <0.01; Figure 2b). In contrast, IDO1 KO mice were not protected from the LPS-induced increase in immobility during the TST (LPS main effect; *F*_1,22_ = 18.29, *P* <0.01; Figure 2c). These data indicate that IDO1 KO mice exhibit a similar behavioral response to LPS as WT mice during the TST. However, IDO1 KO mice do not display reduced preference for sucrose solution implicating brain IDO1 as a critical mediator of the anhedonic response following ICV LPS.

### Intracerebroventricular administration of lipopolysaccharide induces expression of proinflammatory cytokines in the brain at 24 hours, but exploratory locomotor activity is not reduced (Experiment 2B)

**Figure 3:**
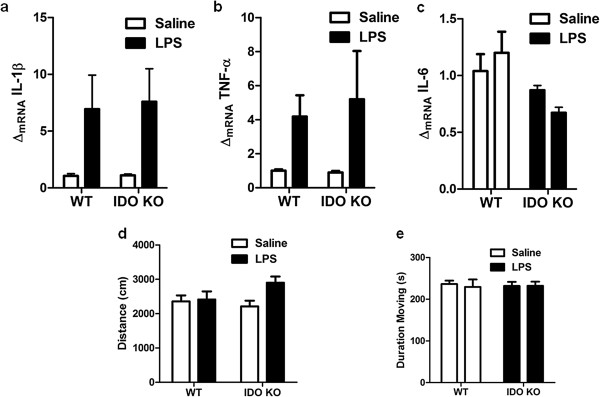
**Brain mRNA expression of proinflammatory cytokines, but not sickness behavior, is increased by intracerebroventricular lipopolysaccharide.** Both wild-type (WT) and indoleamine-2,3-dioxygenase (IDO1) knockout (KO) mice exhibit increased steady-state mRNA expression of (**a**) IL-1β and (**b**) TNFα, but not of (**c**) IL-6 in the whole brain 24 hours post intracerebroventricular (ICV) lipopolysaccharide (LPS, 10 ng). Exploratory locomotor activity measured by (**d**) distance traveled or (**e**) duration of time during the test spent moving were not different 24 hours after ICV LPS. Data are average ± standard error of the mean. *n* = 4 to 5 mice per group.

To determine whether the genotype × LPS interaction on depression-like behavior observed in Figure 2b was a consequence of differential proinflammatory cytokine expression or sickness behavior response, we examined the steady-state expression of IL-1β, TNFα and IL-6 in WT or IDO1 KO mice following ICV LPS or saline administration. Expression of IL-1β (LPS main effect; *F*_1,15_ = 9.71, *P* <0.01; Figure 3a) and TNFα (LPS main effect; *F*_1,15_ = 5.50, *P* <0.05; Figure 3b), but not IL-6 (Figure 3c), was significantly upregulated 24 hours post ICV LPS in both WT mice and IDO1 KO mice. Interestingly, while these proinflammatory cytokines are typically associated with the LPS-induced sickness behavior response, the total distance traveled (Figure 3d) and the duration of time spent moving during the open field test (Figure 3e) was not significantly different between any group 24 hours after ICV LPS administration. Together, these data indicate that the depressive-like behavior of WT and IDO1 KO mice following ICV LPS is not simply a manifestation of a differential recovery from LPS-induced sickness behavior response.

### Central 1-methyl-tryptophan treatment protected mice from central lipopolysaccharide-induced depression-like behavior (Experiment 3)

**Figure 4:**
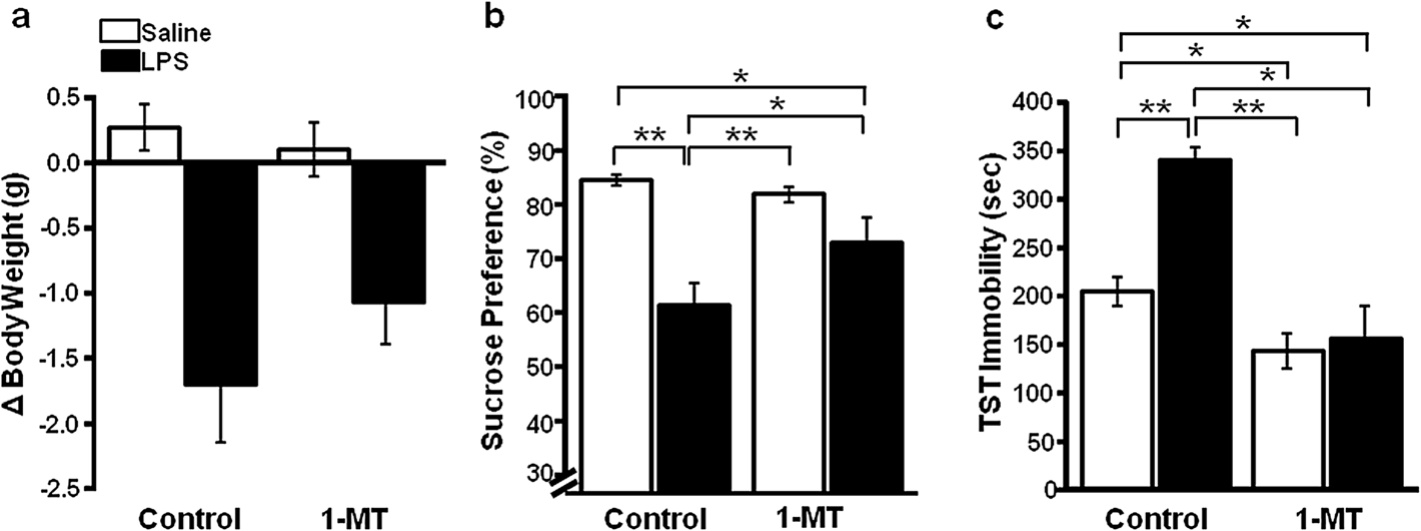
**1-Methyl-tryptophan protects mice from central lipopolysaccharide-induced depression-like behaviors.** (**a**) Mice given 1-methyl-tryptophan (1MT) or saline had similar decreases in body weight in response to central lipopolysaccharide (LPS, 10 ng). (**b**) 1MT-treated mice maintained sucrose preference following intracerebroventricular LPS treatment and (**c**) were protected from LPS-induced increase in tail suspension test (TST) immobility as in wild-type mice. Data are average ± standard error of the mean. *P <0.05, **P <0.01. *n* = 6 mice per group.

To further test our hypothesis that brain IDO1 activity is necessary for developing depression-like behavior in response to ICV LPS, WT C57BL/6J mice were treated with ICV saline or 1MT concurrently with or without LPS. The LPS reduced body weight 24 hours following treatment in both saline mice and 1MT co-treated mice (LPS main effect; *F*_1,21_ = 29.39, *P* <0.01; Figure 4a), indicating that 1MT does not block the nonspecific sickness response. Treatment with 1MT significantly attenuated the anhedonic effects of LPS (1MT × LPS interaction; *F*_3,19_ = 5.18, *P* <0.05; Figure 4b) although LPS decreased the preference for sucrose solution of both saline-treated mice and 1MT-treated mice (LPS main effect; *F*_1,21_ = 26.48, *P* <0.01; Figure 4b). As expected, LPS increased the duration of immobility in the TST in saline-treated control mice (Figure 4c). However, the duration of immobility was unchanged following LPS in mice cotreated with 1MT (1MT × LPS interaction; *F*_3,19_ = 6.43, *P* <0.05). Interestingly, mice that were only treated with 1MT also exhibited reduced TST immobility compared with control + saline-treated mice or control + LPS-treated mice (*P* <0.01). Taken together, these data indicate that inhibiting IDO1 with 1MT does not protect mice from the nonspecific sickness response; however, 1MT protected mice from the development of depression-like behavior following ICV LPS. These data support the hypothesis that IDO1 in the brain is necessary for inducing depression-like behavior following ICV LPS.

## Discussion

Here we show that ICV LPS induced depression-like behavior associated with increased brain kynurenine concentrations. Our data also suggest that brain IDO1 activity is an important regulator of depression-like behavior following ICV LPS, since genetic deletion or pharmacological inhibition of brain IDO1 protected mice from LPS-induced depression-like behavior.

Previous studies established 10 ng LPS as an ICV dose sufficient to induce central IDO1 expression and transient sickness followed by detectable depression-like behaviors [29]. Importantly, we have now demonstrated that this dose of LPS increases IDO1 activity specifically in the brain, as there were no measurable changes in plasma concentrations of kynurenine or tryptophan. Further, ICV LPS induced depression-like behavior concurrent with elevations in brain kynurenine, and IDO1 was required for this behavioral response. Our previous work has demonstrated that IDO1 activation is necessary for inflammation-induced depression-like behavior following systemic immune challenge [7,21,22]. We have also shown that increasing circulating kynurenine levels via exogenous administration of kynurenine to naïve mice is sufficient to induce depression-like behavior [7,22]. However the respective role of peripheral and brain IDO1 in inflammation-induced depression-like behavior was not assessed in these studies.

To determine whether activation of brain IDO1 is required for depression-like behavior, we selected a dose of LPS that, when administered ICV, has no measured effect on peripheral IDO1. We tested the behavioral effect of this dose of LPS in WT mice and in mice whose IDO1 activation was blocked genetically or pharmacologically. In a manner similar to what we previously observed following systemic LPS, ICV LPS elicited a nonspecific sickness response in mice whose IDO1 activation was blocked, as evidenced by body weight loss and upregulation of proinflammatory cytokine expression within the brain. However, IDO1 KO mice maintained sucrose preference following ICV LPS, whereas WT mice exhibited an anhedonia-like response characterized by reduced sucrose preference. In contrast, IDO1 KO mice were not protected from the LPS-induced increase in immobility in the TST, although saline-treated IDO1 KO mice tended to have reduced TST immobility compared with WT mice. The lack of protection observed in IDO1 KO mice during TST testing would indicate that this behavior could be influenced by the induction of other inflammatory mediators acting independently of IDO1 activation.

Evidence exists indicating that cytokines can influence depression-like behaviors independent of IDO1 expression. Our previous research examining the protective effects of insulin-like growth factor (IGF)-I demonstrated that ICV IGF-I, administered prior to LPS, protected against LPS-induced increase in TST immobility [29]. However, when the potential protective benefit of IGF-I was investigated in the sucrose preference test, mice administered IGF-I prior to an LPS challenge were not protected against LPS-induced anhedonia [29]. In these studies, IGF-I decreased central proinflammatory cytokine expression but did not attenuate the induction of brain IDO1 expression. Noteworthy is our previous demonstration that cytokine expression in IDO1 KO mice is not different from that of WT mice following peripheral LPS [7]. These findings suggest that elevated cytokine expression may be necessary to precipitate depression-like behavior in the TST in response to direct neuroimmune challenge, and may be sufficient to drive this behavioral phenotype through an IDO1-independent mechanism. However, activation of the kynurenine pathway may be necessary for central LPS to induce the anhedonic response, as both genetic and pharmacological inhibition of IDO1 mitigated the anhedonia-like effects of LPS.

An interesting paradox emerged in the present dataset; complete genetic deletion of IDO1 protected mice from the behavioral effects of LPS only in the sucrose preference test. Meanwhile, administration of 1MT elicited an antidepressant effect in LPS-treated mice in both the sucrose preference test and the TST. While still speculative, compensatory expression of indoleamine-2,3-dioxygenase 2 (IDO2) or tryptophan 2,3-dioxygenase (TDO2) in the brain might occur in IDO1 KO mice. Both IDO2 and TDO2 divert tryptophan to the kynurenine pathway [35,36]. TDO2 and IDO2 transcript expression is increased in brain tissue following administration of LPS at a time correlated with the presence of depression-like behavior [31]. Moreover, recent evidence has implicated a role of brain TDO2 in anxiety-like behavior because genetic deletion of TDO2 provided anxiolytic effects assessed as an increased time spent in open areas of the elevated plus maze and open field tests [36]. For our model, however, it should be noted that TDO2 activity is not impacted by the presence of 1MT, and therefore it is unlikely that TDO2 is compensating for decreased IDO1 activity and driving depression-like behavior observed in the current studies. IDO2 could perhaps be compensating for the loss of IDO1 in KO mice. In Experiment 3, we utilized the nonselective racemic mixture of 1MT. Although it remains controversial, various studies have demonstrated that levo stereoisomer 1MT provides significant inhibition of IDO1 and IDO2 activity [35], while the dextro stereoisomer 1MT has little effect on IDO1 activity but does inhibit IDO2 activity [35,37]. Even though it is generally accepted that IDO1 has significantly greater enzymatic activity and probably contributes the majority of increased kynurenine in our studies [35,38], our data suggest that an IDO1-independent pathway is responsible for depression-like behavior in the TST following ICV LPS.

The current data are also the first to show that 1MT administered directly into the brain can protect against inflammation-induced depression-like behaviors in a manner comparable with previous studies using systemic 1MT [7,21,22]. Our findings are also in agreement with a recent publication from Dobos and colleagues showing that 1MT reduced time of immobility in the forced swim test four days after a 5 μg dose of ICV LPS was administered [23]. Worthy of note is that the experimental design of Dobos and colleagues’ study was considerably different from the design employed here. The dose of LPS used was 500 times larger than our dose, and the timing of behavioral testing after LPS treatment was not the same. High-dose LPS could cause cell death within the brain, and, as the authors stated, probably invoked peripheral IDO1 activity [23]. Furthermore, we injected 1MT directly into the brain while Dobos and colleagues administered 1MT subcutaneously via a chronic release pellet or via injections without determining inhibitor concentrations within the brain. Our studies were designed to directly investigate the effects of brain IDO1 activity on neuroinflammation-dependent depression-like behavior.

## Conclusions

Taken together, our data confirm that centrally administered LPS induces depression-like behavior that occurs concurrently with elevations in brain kynurenine concentrations. Although additional experiments are necessary to fully determine the role that kynurenine metabolism plays in mediating the behavioral effects of inflammation, our data implicate IDO1 as an important component of central LPS-induced depression-like behavior, specifically sucrose preference. Other dioxygenases with activity similar to IDO1, such as TDO2 and possibly IDO2, could play a role in increasing kynurenine levels in response to central LPS treatment to precipitate certain depression-like behaviors. Depression-like behaviors may be differentially regulated by IDO1-dependent downstream kynurenine metabolism and proinflammatory cytokines; warranting further investigation into these possibilities.

## Abbreviations

HPLC: High-performance liquid chromatography; ICV: Intracerebroventricular; IDO1: Indoleamine-2,3-dioxygenase 1; IDO2: Indoleamine-2,3-dioxygenase 2; IL: Interleukin; KO: Knockout; LPS: Lipopolysaccharide; 1MT: 1-methyl-tryptophan; PBS: Phosphate-buffered saline; PCR: Polymerase chain reaction; RT: Reverse transcriptase; TDO2: Tryptophan 2,3-dioxygenase; TNF: Tumor necrosis factor; TST: Tail suspension test; WT: Wild-type.

## Competing interests

The authors declare that they have no competing interests. JCO and RD have consulted for Lundbeck Research, USA. KWK has received an honorarium from Astra-Zeneca.

## Authors’ contributions

MAL helped to conceive the study, formulated the design of the studies, carried out the execution and analysis of these studies and drafted the manuscript. JMP participated in the execution, analysis and interpretation of the studies and helped draft the manuscript. RHM participated in formulating the design of the studies and interpretation of results and helped to draft the manuscript. RD participated in formulating the design of the studies, interpretation of results and helped to draft the manuscript. KWK participated in formulating the design of the studies, interpretation of results and helped to draft the manuscript. JCO helped to conceive the study, formulated the design of the studies, carried out the execution and analysis of these studies and drafted the manuscript. All authors read and approved the final manuscript.
